# Correlation of psychomotor findings and the ability to partially weight bear

**DOI:** 10.1186/1758-2555-4-6

**Published:** 2012-02-13

**Authors:** Thomas Ruckstuhl, Georg Osterhoff, Michael Zuffellato, Philippe Favre, Clément ML Werner

**Affiliations:** 1 Department of Orthopaedics, University of Zurich, Uniklinik Balgrist, Forchstrasse 340, 8008 Zurich, Switzerland; 2 Division of Trauma Surgery, University Hospital Zurich, Rämistrasse 100, 8091 Zurich, Switzerland; 3 Department of Physical Therapy, University of Zurich, Uniklinik Balgrist, Forchstrasse 340, 8008 Zurich, Switzerland; 4 Department of Biomechanics, University of Zurich, Uniklinik Balgrist, Forchstrasse 340, 8008 Zurich, Switzerland

**Keywords:** Partial weight bearing, Psychomotor testing, Rehabilitation

## Abstract

**Background:**

Partial weight bearing is thought to avoid excessive loading that may interfere with the healing process after surgery of the pelvis or the lower extremity. The object of this study was to investigate the relationship between the ability to partially weight bear and the patient's psychomotor skills and an additional evaluation of the possibility to predict this ability with a standardized psychomotor test.

**Methods:**

50 patients with a prescribed partial weight bearing at a target load of 15 kg following surgery were verbally instructed by a physical therapist. After the instruction and sufficient training with the physical therapist vertical ground reaction forces using matrix insoles were measured while walking with forearm crutches. Additionally, psychomotor skills were tested with the Motorische Leistungsserie (MLS). To test for correlations Spearman's Rank correlation was used. For further comparison of the two groups a Mann-Withney test was performed using Bonferroni correction.

**Results:**

The patient's age and body weight significantly correlated with the ability to partially weight bear at a 15 kg target load. There were significant correlations between several subtests of the MLS and ground reaction forces measured while walking with crutches. Patients that were able to correctly perform partial weight bearing showed significant better psychomotor skills especially for those subtests where both hands had to be coordinated simultaneously.

**Conclusions:**

The ability to partially weight bear is associated with psychomotor skills. The MLS seems to be a tool that helps predicting the ability to keep within the prescribed load limits.

## Background

Partial weight bearing is commonly prescribed after surgery on a lower extremity. It restricts weight bearing on the affected leg to avoid excessive loading that may result in a prolonged healing period or even a stop of the healing process [1-3]. It would be important to detect patients unable to accurately partially weight bear. Knowing in advance which patients were expected to overload the extremity would enable the surgeon to choose a different procedure with a more stable fixation or prescribe a different rehabilitation program. In several preceding studies it could be shown that patients are unable to control the load on the involved side as prescribed [2-6]. Chow et al. [1] found that muscle power of the contralateral extremity and the mental state influence the ability to partially weight bear. They also reported that the left hand grip was the most significant predictive value for the partial weight bearing ability followed by the mental state. It is, however, still not established to predict the patient's ability to partially weight bear before surgery. The described correlation between the left hand grip and effective partial weight bearing by Chow et al. [1] might indicate a connection between the psychomotor skills of the patient and the ability to partially weight bear. Previously, a correlation has been shown between the results in a psychomotor test battery (Motorische Leistungsserie, MLS) [7,8] and the clinical outcome after tendon transfers or in patients with scapular dyskinesis [9,10]. While the reliability and reproducibility of the left hand grip might be strongly tester-dependent, the MLS provides a standardized and validated procedure [7,8] that is easy to handle at low costs. If the ability to partially weight bear was associated with individual psychomotor skills, psychomotor testing with the MLS could be used and established to identify patients at risk preoperatively.

Thus, a prospective clinical study was designed to test for potential interrelations between the psychomotor level and the force generated from partial weight walking. It was our hypothesis that patients with good psychomotor skill will rather be able to perform partial weight bearing correctly.

## Methods

Between May and August 2008 50 consecutive patients with a prescribed partial weight bearing at a 15 kg target load were tested. The average age was 46.5 years with a range from 16 to 83 years of age. The mean body weight was 75.5 kg ranging from 45 to 112 kg. The inclusion criterion was a prescribed partial weight bearing at a 15 kg target load regardless of the procedure the patient underwent. Exclusion criteria included an additional injury at the upper extremity, additional medical problems not allowing testing, or a seriously altered mental state making an accurate instruction for partial weight bearing impossible. This study has been approved by the institutional review board and a written informed consent was obtained from all patients before they were included in the study. After surgery the patients were instructed to partially weight bear on forearm crutches by members of the department of physical therapy. Patients were introduced to partial weight bearing using parallel bars with visual feedback for the patient by an analogue scale. When patients were able to carry out proper flexing action during walking, they were instructed in using crutches. As soon as the therapist confirmed that the patient had seemed repeatedly to be able to partially weight bear by using an analogue scale, a psychomotor test and the measurements of ground reaction forces were performed as described below. The number of days from the first mobilization until the patient was cleared by the physical therapist was recorded. To avoid distortion of the results by the educational level as it might be expected due to different comprehension of the physiotherapists explanations or a different knowledge of the muskulo-skeletal anatomy, it was determined and divided into five classes (1 = no graduation; 2 = minimum of 9-10 years of school with graduation; 3 = vocational school with up to 12 years of education; 4 = general qualification for university entrance and 12-13 years of education; 5 = university degree).

### Psychomotor skill testing

The patient's psychomotor skills were tested with the standard version of the Motorische.

**Figure 1:**
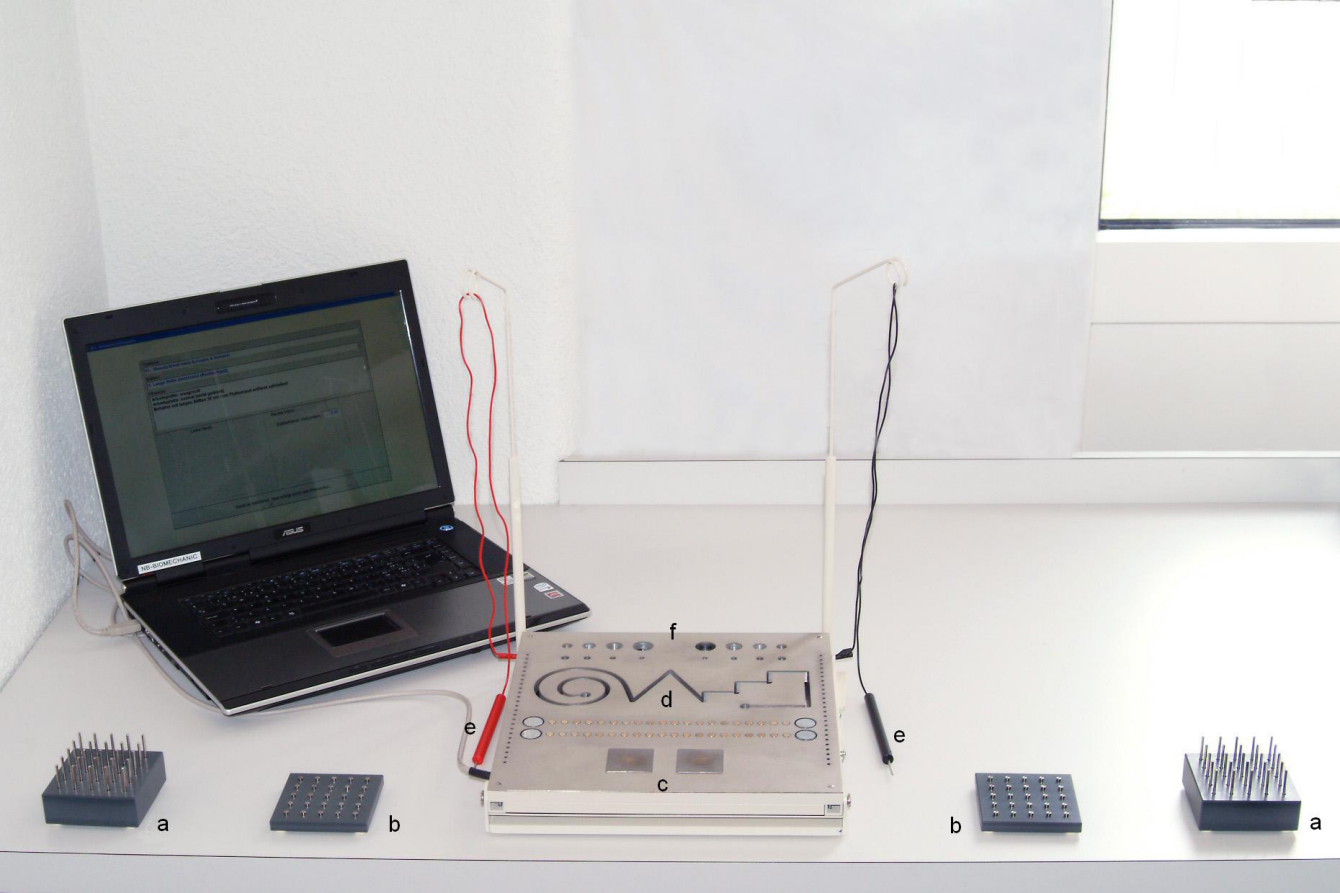
**Motorische Leistungserie (MLS)**. Illustration of the Motorische Leistungsserie (MLS) consisting of a working platform with long (**a**) and short (**b**) pins as well as electrical contacts to test tapping frequency (**e **+ **c**), aiming and steadiness (**e **+ **f**) and line tracking ability (**e **+ **d**). The platform is connected to a personal computer with the corresponding software for test analysis.

Leistungsserie (MLS) (Wiener Testsystem, Schuhfried GmbH, Mödling, Austria) described by Schoppe [8] and Hamster [7]. This test consists of a working platform with different reamings, pins and electrical contacts and is connected to a personal computer with the corresponding software for test analysis (Figure 1). The standard psychomotor skill test [8] is composed of 21 subtests performed with each, the dominant and the non-dominant, hand. Additionally, five of these subtests are carried out with both hands simultaneously. The results of all 21 subtests are then evaluated for each, the dominant and the non-dominant, upper extremity separately and are correlated to normative values from healthy individuals by the software included in the test kit.

### Force measurements

**Figure 2:**
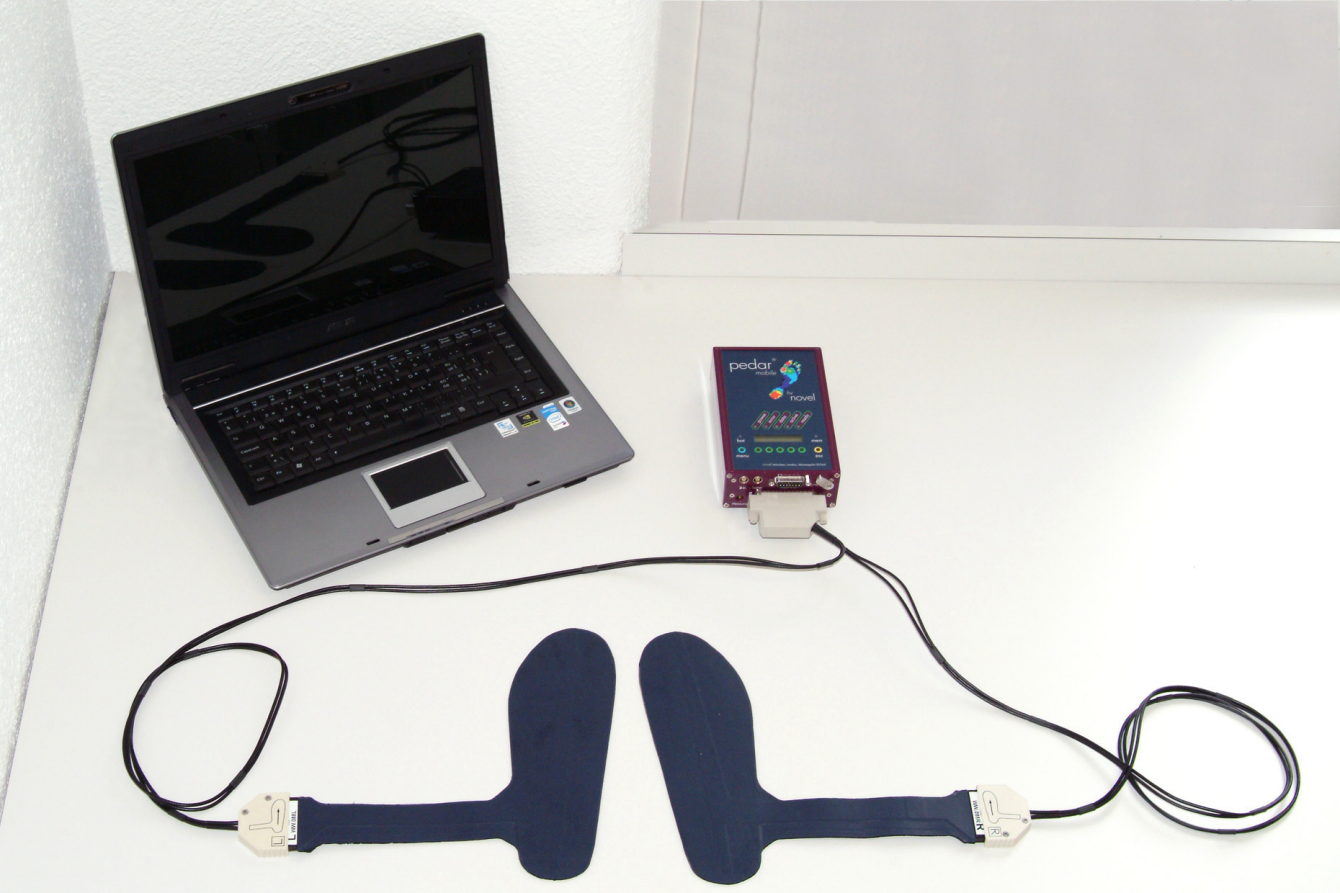
**Pedar mobile system**. Illustration of the Pedar Mobile system with the matrix insoles that are connected to the recording device. This device is further connected to a personal computer with the respective software to evaluate the measurements.

The load on the lower extremity was measured with the Pedar Mobile system (version 8.2; Novel GmbH, Munich, Germany) (Figure 2). The matrix insoles of this portable device contain 99 capacitance sensors to detect and measure vertical forces during walking. Calibration of the insoles was carried out with a Trublu calibration device (Novel GmbH, Munich, Germany) according to the manufacturer's guidelines. Insoles of appropriate size were placed in the shoes of the patient and connected to the recording device which itself was connected to a personal computer. After doing zero settings of the instruments patients were asked to walk a distance of about 20 m on forearm crutches without help while the insoles recorded the vertical forces. Maximal load (Fmax) for each step was evaluated with the analysis software of the test system and the average maximal force (average Fmax) was calculated. To measure how constant the patients loaded their extremities the standard deviation of Fmax (SD Fmax) was determined. Since it was thinkable that differences in weight bearing simply were caused by differences in total body weight, the average Fmax was expressed as the percentage of the patient's body weight, additionally. Arbitrarily, a cut-off was set at 180 N to subdivide the population into group 1 (Fmax < = 180 N) complying with the prescription and group 2 (Fmax > 180 N) overloading the extremity.

### Statistics

Statistical analysis was performed by a consultant using SPSS 13 for Windows (SPSS Inc., Chicago, Illinois). To test for correlations Spearman's Rank correlation was used. For comparison of the two groups a Mann-Whitney test was performed and ROC curves were calculated. A Bonferroni correction for multiple comparisons was performed. As the different subtests of the MLS testing battery do not represent independent items and to avoid a possible overcorrection towards an overrated Type II error, adjustment was performed for the five hypothesis-deduced main items (i.e. psychomotor skills of the right hand, psychomotor skills of the left hand, psychomotor skills of both hands simultaneously, age, body weight) [11,12]. Thus, with the Type I error set at 0.05, differences were considered significant for *p *≤ 0.05/5 = 0.01 [12].

## Results

The average Fmax calculated for all patients was 177 N (range 18.5-569.9 N), the mean SD Fmax was 40.77 N (range 8.62-88.4 N). The average percentage of the patient's body weight was 23.7% (range 2.4-77.1%). On average patients needed 2.88 days (range 1-11 days) to learn to partially weight bear. All but one patient were right-handed.

A significant correlation could be found between the average Fmax and the age of the patient (*p *= 0.001, r = 0.462) as well as with the patient's body weight (*p *= 0.003, r = 0.416). Several subtests of the MLS correlated significantly with the average Fmax. Steadiness, inserting short and long pins correlated positive with the average Fmax when tested on the right, the left and on both hands simultaneously. The total duration of the subtest line tracking (tested on the right and on the left) correlated negatively with the average Fmax. For the number of hits in the subtest tapping, a negative correlation with Fmax could be shown when tested on both sides individually as well as simultaneously (Table 1).

**Table 1:** Significant correlations of psychomotor skills with Fmax and SD Fmax

Parameter	Mean	Minimum	Maximum	Correlation to Fmax*	Correlation to SD Fmax*
**Fmax **[N]	177	18.5	569.9		
**SD Fmax **[N]	40.8	8.6	88.4		

**Age**	46.5	16	83	**.462**	.335
**Body weight **[N]	741	441	1098	**.416**	.288

aiming errors **right**	1.1	0	5	**-.121**	-.142
steadiness errors **right**	22.2	1	120	**.464**	.240
steadiness error duration **right **[s]	3.60	0.01	21.17	**.502**	.251
line tracking total duration **right **[s]	29.11	8.88	79.57	**-.422**	-.310
inserting long pins **right **[s]	50.15	33.80	83.10	**.403**	.352
inserting short pins **right **[s]	54.64	36.19	100.48	**.422**	.341
tapping **right**	179.3	120	234	**-.423**	-.317

steadiness errors **left**	27.5	1	127	**.432**	.217
steadiness error duration **left **[s]	2.90	0.01	16.65	**.424**	.252
line tracking total duration **left **[s]	27.07	7.85	90.79	**-.476**	-.297
inserting long pins **left **[s]	53.46	38.37	105.65	**.407**	.306
tapping **left**	160.3	80	216	**-.391**	-.245

steadiness errors right **bh**	26.7	2	95	**.432**	.207
steadiness error duration right **bh **[s]	5.46	0.13	28.34	**.434**	.287
steadiness error duration left **bh **[s]	6.38	0.36	25.16	**.413**	**.407**
inserting long pins right **bh **[s]	80.31	49.04	147.45	**.447**	**.401**
inserting long pins left **bh **[s]	80.63	50.80	146.68	**.422**	**.387**
inserting short pins right **bh **[s]	88.93	54.70	160.94	**.493**	**.395**
inserting short pins left **bh **[s]	89.83	56.89	157.78	**.478**	.376
tapping right **bh**	156.6	48	229	**-.563**	**-.464**
tapping left **bh**	151.9	61	217	**-.429**	-.359

**CC**: Correlation Coefficient, **bh**: both hands simultaneously

*Bold numbers: *p *< 0.01

In contrast to the average Fmax, the SD Fmax only showed significant correlation with several subtests of the MLS when tested on both hands simultaneously (Table 1).

No association could be found between the average Fmax and the number of days needed to learn to partially weight bear, sex, education and affected leg.

For further statistical analysis the population was divided into two groups as described above.

As expected, SD max differed noticeably between both groups. Patients in group 1 (40.1 years) were younger than in group 2 (55.3 years) even though this was statistically not significant (*p *= 0.013). Several parameters of the MLS subtests showed significant differences between the two groups when tested on each side separately (Table 2). Again, when tested on both sides simultaneously, differences between the two groups became even more evident (Table 2).

**Table 2:** Significant differences of psychomotor skills between Group 1 (Fmax = < 180 N) and Group 2 (Fmax > 180 N)

Parameter	Mean	Mean	p*
	Group 1	Group 2	
**Fmax **[N]	96.4	288.4	**<.001**
**SD Fmax **[N]	33.0	51.5	**.001**

**Percentage body weight**	13.9	37.3	**<.001**

**Age**	40.1	55.3	.013
**Body weight **[N]	696	802	.026

aiming errors **right**	13.7	34.0	**.002**
aiming error duration **right **[s]	1.75	6.15	**.001**
inserting short pins **right **[s]	49.02	62.91	**.004**

steadiness errors **left**	18.30	40.10	**.003**
line tracking total duration **left **[s]	31.42	21.06	**.005**
inserting short pins **left **[s]	55.20	71.09	**.007**

aiming errors right **bh**	1.3	3.5	**.004**
aiming error duration right **bh **[s]	0.18	0.46	**.006**
steadiness errors right **bh**	20.5	35.8	**.002**
steadiness error duration right **bh **[s]	3.69	8.02	**.007**
inserting short pins right **bh **[s]	79.40	103.75	**.006**
inserting short pins left **bh **[s]	80.44	104.45	**.006**
tapping right **bh**	167.9	140.2	**.001**
tapping left **bh**	162.6	136.5	**.004**

**CC**: Correlation Coefficient, **bh**: both hands simultaneously

*Bold numbers: *p *< 0.01

The results of all single tested parameters are accessible as Additional file 1: Table S1 and Additional file 2: Table S2.

## Discussion

Even though some authors found it possible to partially weight bear according to the prescribed limits the majority of studies showed that patients were not able to keep within the given load [2-5]. All studies that we are aware which reported the ability to accurately follow the instructions were performed with healthy subjects [6,13,14]. Therefore, their results can not accurately be assigned to patients since their inability to partially weight bear must be assumed. Chow et al. [1] reported that the patient's potential to partially weight bear was predictable by simply testing the left hand grip and performing a mental state test. Based on these findings we hypothesized that the ability to partially weight bear is associated with the psychomotor skills of the patient and therefore predictable with a motor performance test. Such a relationship should be searched for in this study. Statistical analysis showed a significant correlation of the average Fmax and the age as well as the patient's body weight. This disagrees with Chow et al. [1] who reported no significance for these factors. The evaluation of the results of several subtests of the MLS showed a significant correlation with the average Fmax. It was thereby not important whether the tests were performed on the right, the left or on both sides simultaneously. Subtests correlating with the SD Fmax could be used to predict how constant the patient is able to partially weight bear.

The total duration of the subtest line tracking and the number of hits of the subtest tapping seemed to be especially dependent on the compliance and could therefore be an indicator to determine who will take the instructions seriously. Since complying with the therapist's instructions is crucial for accurate partial weight bearing these tests might be very useful.

Comparing the two groups with each other showed that the results of several subtests differed significantly which indicates the possibility to predict the ability to partially weight bear. It is of importance, that patients in Group 2 not only loaded the concerned leg with Fmax > 180 N but also with a higher percentage of their own body weight and had an increased SD Fmax. This indicates that correct partial weight bearing is rather a matter of psychomotor control than of body mass.

Furthermore, a regression analysis was performed trying to calculate the average Fmax using the parameters of the MLS. The resulting formula with ten different factors turned out to be too complicated and arguable for clinical use.

The Pedar Mobile system used for force measurements has been validated by Hurkmans et al. [15,16]. But there are still some problems associated with this system that have to be considered as limiting factors. The sensors of the insoles have a threshold value to minimize confounders. Forces below these values are not registered and therefore the measurements are slightly to low. Since this threshold value was the same for all patients comparison of the two groups was not influenced. The relation between the average Fmax and parameters of the MLS are also not affected because the loss of force is small and assumed about equal for all patients. Falsification of results caused by this confounder is therefore within reasonable limits. A small amount of force is further directed via the shoe and not via the insole to the ground resulting in an additional loss of force. To minimize these confounders Fong et al. [17] described a method to estimate the complete ground reaction force with pressure insoles. The described technique could not be adapted to this investigation because patients often showed an altered moving pattern loading only some parts of the insoles.

For this study, only short-term force measurements in presence of an investigator have been performed. It has to be assumed that patients are taking more care in partial weight bearing when accompanied than when unobserved. Since this study was designed to investigate the ability to accurately partially weight bear and to search for an association to the patient's psychomotor skills short-term measurements were sufficient. Since measurements were performed only a few days after surgery, pain could have been a further confounding factor leading to more accentuated unloading of the extremity. To minimize this effect, patients were only tested when they declared no pain. For organizational reasons psychomotor skills were tested after surgery. A certain influence of medication on the test results could not be excluded. To establish equal conditions all patients were tested postoperatively and the test was not performed when the patient was obviously influenced by medication.

Eventually, the patients' absolute strength or endurance of the upper limbs could have influenced their ability of correct partial weight bearing. We did, however, not consider the maximum load of each patient but averaged Fmax on a rather short walking distance of 20 m, thus minimizing the relevance of strength endurance. Additionally, it is likely that lowered absolute upper extremity strength has direct implications on the results of the MLS that is operated by using arms, hands and fingers.

Some authors state that motor control resources are specific and that transfer between skills is small [18,19]. Thus, at first sight, a method specifically measuring the psychomotor skills of the lower extremities might seem favourable to draw conclusions on the ability of intentionally decreasing the load on one leg. Partial weight bearing on forearm crutches, however, is a complex interaction of all four extremities and the trunk. Finally, testing protocols involving the legs are just not applicable for patients with injuries of the lower extremities. Our results showed differences between Group 1 ("good" partial weight bearing) and Group 2 ("bad" partial weight bearing) especially for those subtests where both hands had to be coordinated simultaneously as this would be required for walking on crutches.

The key question of this study was whether some persons have more ability than others to coordinate the muscles of their trunk, arms and legs and adapt it to a new weight bearing situation. We, therefore, decided to use a standardized and validated procedure testing general psychomotor skills [7,8]--the MLS.

## Conclusions

An association between the ability to partially weight bear and psychomotor skills could be shown in this study. Patients with better psychomotor skills have a higher propability of being able to correctly perform partial weight bearing. This accounts in particular for those subtests of the MLS that have to be done with both hands simultaneously. We therefore assume that it is possible to use the MLS as a tool to predict the ability to partially weight bear. Before clinical use threshold values for the subtests have to be determined with more patients. Since the MLS is a test that can be performed very easily, clinical use to predict the ability to comply with the prescribed load is realistic.

## Competing interests

The authors declare that they have no competing interests.

## Authors' contributions

TR participated in the study design, carried out all measurements, participated in the statistical analysis and drafted the manuscript. GO revised the statistical analysis and the manuscript. MZ participated in the study design and assisted in the measurements. PF participated in the study design and assisted in the measurements. CW conceived of the study and participated in its design, participated in the statistical analysis and helped to draft the manuscript. All authors read and approved the final manuscript.

## Supplementary Material

Additional file 1 **Table S1**. Correlations of psychomotor skills with Fmax and SD Fmax-all results. Click here for file

Additional file 2 **Table S2**. Differences of psychomotor skills between Group 1 and Group 2-all results. Click here for file
