# Supplementary material for: Correlation of psychomotor findings and the ability to partially weight bear
Source: Sports Med Arthrosc Rehabil Ther Technol. 2012 Feb 13;4:6. doi: 10.1186/1758-2555-4-6 (PMC3307441; doi:10.1186/1758-2555-4-6)
Supplement: Additional file 1 — Table S1. Correlations of psychomotor skills with Fmax and SD Fmax-all results. [file 1758-2555-4-6-S1.DOC]

| **Parameter** | **Mean** | **Minimum** | **Maximum** | **Correlation to**  **Fmax** | **Correlation to**  **SD Fmax** |
| --- | --- | --- | --- | --- | --- |
|  |  |  |  |  |  |
| **Fmax** [N] | 177 | 18.5 | 569.9 |  |  |
| **SD Fmax** [N] | 40.8 | 8.6 | 88.4 |  |  |
|  |  |  |  |  |  |
| **Age** | 46.5 | 16 | 83 | .462 | .335 |
| **Body weight** [N] | 741 | 441 | 1098 | .416 | .288 |
|  |  |  |  |  |  |
| aiming errors **right** | 1.1 | 0 | 5 | -.121 | -.142 |
| aiming error duration **right** [s] | .65 | .00 | .42 | -.144 | -.154 |
| aiming total duration **right** [s] | 10.80 | 5.98 | 27.34 | .261 | .197 |
| steadiness errors **right** | 22.2 | 1 | 120 | .464 | .240 |
| steadiness error duration **right** [s] | 3.60 | 0.01 | 21.17 | .502 | .251 |
| line tracking errors **right** | 28.8 | 7 | 107 | .336 | .191 |
| line tracking error duration **right** [s] | 3.29 | 0.17 | 20.98 | .356 | .127 |
| line tracking total duration **right** [s] | 29.11 | 8.88 | 79.57 | -.422 | -.310 |
| inserting long pins **right** [s] | 50.15 | 33.80 | 83.10 | .403 | .352 |
| inserting short pins **right** [s] | 54.64 | 36.19 | 100.48 | .422 | .341 |
| tapping **right** | 179.3 | 120 | 234 | -.423 | -.317 |
|  |  |  |  |  |  |
| aiming errors **left** | 2.2 | .00 | 9 | .086 | .043 |
| aiming error duration **left** [s] | .14 | .00 | .80 | -.011 | -.009 |
| aiming total duration **left** [s] | 10.90 | 7.24 | 17.67 | .279 | .158 |
| steadiness errors **left** | 27.5 | 1 | 127 | .432 | .217 |
| steadiness error duration **left** [s] | 2.90 | .01 | 16.65 | .424 | .252 |
| line tracking errors **left** | 33.3 | 12 | 97 | -.075 | -.137 |
| line tracking error duration **left** [s] | 3.85 | .88 | 12.21 | .266 | .088 |
| line tracking total duration **left** [s] | 27.07 | 7.85 | 90.79 | -.476 | -.297 |
| inserting long pins **left** [s] | 53.46 | 38.37 | 105.65 | .407 | .306 |
| inserting short pins **left** [s] | 61.69 | 40.76 | 114.57 | .347 | .213 |
| tapping **left** | 160.3 | 80 | 216 | -.391 | -.245 |
|  |  |  |  |  |  |
| aiming errors right **bh** | 2.2 | 0 | 18 | .259 | -.056 |
| aiming errors left **bh** | 7.5 | 0 | 24 | .243 | .110 |
| aiming error duration right **bh** [s] | .30 | .00 | 2.22 | .286 | .040 |
| aiming error duration left **bh** [s] | 1.12 | .00 | 8.68 | .266 | .056 |
| aiming total duration right **bh** [s] | 17.25 | 9.28 | 32.17 | .234 | .140 |
| aiming total duration left **bh** [s] | 17.09 | 9.32 | 32.17 | .285 | .199 |
| steadiness errors right **bh** | 26.7 | 2 | 95 | .432 | .207 |
| steadiness errors left **bh** | 34.6 | 6 | 92 | .354 | .350 |
| steadiness error duration right **bh** [s] | 5.46 | 0.13 | 28.34 | .434 | .287 |
| steadiness error duration left **bh** [s] | 6.38 | 0.36 | 25.16 | .413 | .407 |
| inserting long pins right **bh** [s] | 80.31 | 49.04 | 147.45 | .447 | .401 |
| inserting long pins left **bh** [s] | 80.63 | 50.80 | 146.68 | .422 | .387 |
| inserting short pins right **bh** [s] | 88.93 | 54.70 | 160.94 | .493 | .395 |
| inserting short pins left **bh** [s] | 89.83 | 56.89 | 157.78 | .478 | .376 |
| tapping right **bh** | 156.6 | 48 | 229 | -.563 | -.464 |
| tapping left **bh** | 151.9 | 61 | 217 | -.429 | -.359 |
|  |  |  |  |  |  |
| **CC**: Correlation Coefficient |  |  | |  |  |
| **bh**: both hands simultaneously |  |  |  |  |  |
