# Supplementary material for: Correlation of psychomotor findings and the ability to partially weight bear
Source: Sports Med Arthrosc Rehabil Ther Technol. 2012 Feb 13;4:6. doi: 10.1186/1758-2555-4-6 (PMC3307441; doi:10.1186/1758-2555-4-6)
Supplement: Additional file 2 — Table S2. Differences of psychomotor skills between Group 1 and Group 2-all results. [file 1758-2555-4-6-S2.DOC]

| **Parameter** | **Mean**  **Group 1** | **Mean**  **Group 2** | **p** |
| --- | --- | --- | --- |
|  |  |  |  |
| **Fmax** [N] | 96.4 | 288.4 | <.001 |
| **SD Fmax** [N] | 33.0 | 51.5 | .001 |
| **Percentage body weight** | 13.9 | 37.3 | <.001 |
| **Age** | 40.1 | 55.3 | .013 |
| **Body weight** [N] | 696 | 802 | .026 |
|  |  |  |  |
| aiming errors **right** | 13.7 | 34.0 | .002 |
| aiming error duration **right** [s] | 1.75 | 6.15 | .001 |
| aiming total duration **right** [s] | 9.97 | 11.94 | .212 |
| steadiness errors **right** | 24.6 | 34.7 | .040 |
| steadiness error duration **right** [s] | 2.45 | 4.47 | .027 |
| line tracking errors **right** | 32.18 | 24.87 | .024 |
| line tracking error duration **right** [s] | 46.86 | 54.70 | .023 |
| line tracking total duration **right** [s] | 24.6 | 34.7 | .040 |
| inserting long pins **right** [s] | 2.45 | 4.47 | .027 |
| inserting short pins **right** [s] | 49.02 | 62.91 | .004 |
| tapping **right** | 186.8 | 168.9 | .024 |
|  |  |  |  |
| aiming errors **left** | 1.9 | 2.7 | .301 |
| aiming error duration **left** [s] | .12 | .18 | .646 |
| aiming total duration **left** [s] | 10.29 | 11.75 | .128 |
| steadiness errors **left** | 18.30 | 40.10 | .003 |
| steadiness error duration **left** [s] | 2.02 | 4.18 | .010 |
| line tracking errors **left** | 32.0 | 35.1 | .859 |
| line tracking error duration **left** [s] | 3.28 | 4.64 | .074 |
| line tracking total duration **left** [s] | 31.42 | 21.06 | .005 |
| inserting long pins **left** [s] | 49.92 | 58.36 | .030 |
| inserting short pins **left** [s] | 55.20 | 71.09 | .007 |
| tapping **left** | 168.0 | 149.7 | .021 |
|  |  |  |  |
| aiming errors right **bh** | 1.3 | 3.5 | .004 |
| aiming errors left **bh** | 6.5 | 9.1 | .046 |
| aiming error duration right **bh** [s] | 0.18 | 0.46 | .006 |
| aiming error duration left **bh** [s] | 0.88 | 1.48 | .029 |
| aiming total duration right **bh** [s] | 16.04 | 19.01 | .120 |
| aiming total duration left **bh** [s] | 15.81 | 18.94 | .084 |
| steadiness errors right **bh** | 20.5 | 35.8 | .002 |
| steadiness errors left **bh** | 30.2 | 41.0 | .052 |
| steadiness error duration right **bh** [s] | 3.69 | 8.02 | .007 |
| steadiness error duration left **bh** [s] | 4.86 | 8.57 | .042 |
| inserting long pins right **bh** [s] | 73.75 | 89.83 | .010 |
| inserting long pins left **bh** [s] | 74.65 | 89.29 | .017 |
| inserting short pins right **bh** [s] | 79.40 | 103.75 | .006 |
| inserting short pins left **bh** [s] | 80.44 | 104.45 | .006 |
| tapping right **bh** | 167.9 | 140.2 | .001 |
| tapping left **bh** | 162.6 | 136.5 | .004 |
|  |  |  |  |
| **CC**: Correlation Coefficient |  |  |  |
| **bh**: both hands simultaneously |  |  |  |
